# Heritability of neonatal acute phase protein levels

**DOI:** 10.1016/j.bbih.2025.101097

**Published:** 2025-09-12

**Authors:** Hugo Sjöqvist, Martin Brynge, Kristiina Tammimies, Ralf Kuja-Halkola, Sven Bölte, Christina Dalman, Renee M. Gardner, Håkan Karlsson

**Affiliations:** a Department of Global Public Health, Karolinska Institutet, Stockholm, Sweden; b Department of Clinical Neuroscience, Division of Psychiatry, Karolinska Institutet, Stockholm, Sweden; c Center of Neurodevelopmental Disorders (KIND), Department of Women's and Children's Health, Centre for Psychiatry Research, Karolinska Institutet & Region Stockholm, Stockholm, Sweden; d Department of Highly Specialized Pediatric Orthopedics and Medicine, Astrid Lindgren Children's Hospital, Karolinska University Hospital, Region Stockholm, Stockholm, Sweden; e Department of Medical Epidemiology and Biostatistics, Karolinska Institutet, Stockholm, Sweden; f Child and Adolescent Psychiatry, Stockholm Health Care Services, Region Stockholm, Stockholm, Sweden; g Curtin Autism Research Group, Curtin School of Allied Health, Curtin University, Perth, Australia; h Department of Neuroscience, Karolinska Institutet, Stockholm, Sweden

**Keywords:** Autism, Acute phase proteins, Twins, ACE, Heritability, Environmental, Neonatal period, Inflammation

## Abstract

Levels of neonatal Acute Phase Proteins (APPs) have been associated with autism and schizophrenia. The relative contributions of genetic and environmental factors to variation in APP levels in the neonatal period are not known. Therefore, we used one of the largest twin samples to date to map the proportions of heritable and non-heritable factors to variations in APPs measured shortly after birth. Moreover, we investigated if any association existed between neonatal APP levels and autism, among monozygotic and dizygotic twins discordant for autism.

Twins were identified and enrolled from registers and a clinical twin study of autism in Sweden. The distributions of APPs measured in dried blood spots taken a few days after birth as part of a national screening program were standardized to reduce any analytical artifacts. The additive genetic (A), common (C) and unique (E) environment components were estimated, using the ACE model, on a sample of 92 twin pairs. We included 61 autism discordant twin pairs for estimating the association between the APPs and autism, using both non-fixed (between) and fixed (within) effects regression models.

For the ACE models, variations in α-2 macroglobulin, C-reactive protein, ferritin, fibrinogen, haptoglobin, serum amyloid A and serum amyloid P were all largely explained by additive genetic factors (70–90 %). Variation in tissue plasminogen activator and procalcitonin were predominantly explained by common environmental factors (60–70 %) with a negligible contribution by genetic factors. Variations in levels of tissue plasminogen activator and procalcitonin in the neonatal period appear to be mainly explained by pregnancy and birth related factors. Variations in levels of other investigated acute phase proteins were largely explained by additive genetic factors. None of the APPs exhibited any significant association with autism in discordant mono- or dizygotic twin pairs. Our findings highlight the importance of considering potential familial confounding in future studies of association between any APP and autism.

## Highlights

• This is the first twin study of neonatal Acute Phase Proteins (APPs).
• Seven out of nine investigated neonatal APPs appeared highly heritable.
• Tissue plasminogen activator levels are less heritable than reported adult levels.
• Levels of procalcitonin appeared mainly influenced by the perinatal environment.
• None of the APP levels were associated with autism, among discordant twins.

## 1 Introduction

Acute Phase Proteins (APPs) are effector molecules of the innate immune system. Variation in levels of APPs measured shortly after birth have previously been associated with autism and schizophrenia (Gardner et al., 2013, 2021). Observed variations in adult APP levels appear to be explained by additive genetic and unique environmental components. We have previously observed that variation in neonatal levels of APPs is associated with maternal factors like length of pregnancy and maternal age (Gardner et al., 2021) indicating a potentially large contribution by shared environmental components to variation in the neonatal period. No study to date has, however, examined the relative contributions of heritable and non-heritable factors to variations in APP levels in early life.

In our previous study (Gardner et al., 2021), the associations observed in analyses of unrelated individuals assessing APP levels at birth and odds of autism differed in the analyses of siblings compared to analyses using unrelated controls (Gardner et al., 2021). Sibling comparison regression models account for confounding factors shared between siblings, e.g., on average 50 % of common genetic variation, suggesting that associations between neonatal APP levels and odds of autism observed in the general population of unrelated individuals may have been confounded by factors shared between siblings. The potential for any residual confounding by pregnancy-related factors remains an open question as siblings do not share their intrauterine environments.

Here, our primary aim was to estimate the relative contributions of genetic and environmental components to variation in neonatal APP levels, using the differences observed in the correlations within monozygotic and dizygotic twin pairs. By doing so, we can estimate the proportions of variation that can be explained by heritable (genetic) factors and by non-heritable factors (e.g. common and/or unique environmental factors), respectively.

A secondary aim was to investigate if any associations between neonatal APP levels and later autism can be observed among twins. By restricting the sample to twin pairs, it is possible to account for any potential residual unmeasured confounding by several pregnancy-related factors as twins share many aspects of the intrauterine environment.

## 2 Material and methods

### 2.1 Study population

Twins were sampled in two different steps.

The first subsample came from the project Roots of Autism and Attention-Deficit/Hyperactivity Disorder (ADHD) Twin Study in Sweden (RATSS) (Myers et al., 2021; Bölte et al., 2014), where recruitment from twin pairs in Sweden (until February 2017) was done for twins above the age of 8 years. Twin pairs, where at least one twin was diagnosed with a neurodevelopmental condition (autism or ADHD), were recruited along with neurotypical twin pairs. Zygosity was determined either by DNA testing, or through a questionnaire focused on physical appearance (Stamouli et al., 2018; Isaksson et al., 2022). For inclusion in this study, both twins were required to have an available neonatal dried bloodspot (NDBS) stored in the national biobank (see 2.3 below). Included twin pairs were born in Sweden during 1985–2008.

The second subsample was identified during our previous collection based on the Stockholm Youth Cohort (SYC) (Idring et al., 2012, 2015), as detailed previously (Gardner et al., 2021). All children with autism (diagnosed up to 31 December 2011) and a random sample of unaffected children born between 1996 and 2000 (Gardner et al., 2021) were identified. In the case of twin births, NDBS samples were collected from both twins and set aside for this study. Children in the SYC subsample had a requirement of living in Stockholm for at least four years to ensure sufficient follow-up time to allow for case ascertainment. An updated ascertainment was performed after sample collection, scanning the already selected sample for autism diagnoses up to 31 December 2016 (Gardner et al., 2021). Zygosity status was obtained from the Swedish Twin Registry.

**Fig. 1:**
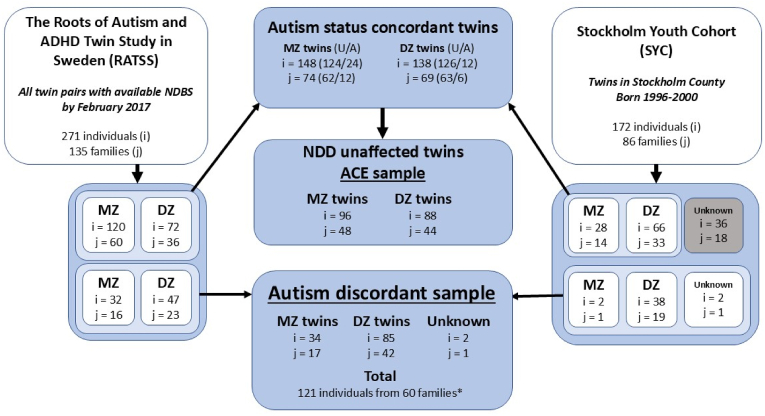
Flowchart of the twins from the two different data sources. NDBS=Neonatal dried bloodspots; MZ = Monozygotic; DZ = Dizygotic; NDD=Neuro Developmental Disorder; U/A = Unaffected/Affected. One family of the autism discordant dizygotic twin pairs had triplets, which were later remade into two families with the autistic child being duplicated for the two controls, i.e. 122 individuals from 61 families. One twin pair had a missing value for A2M, CRP, HAP, and SAP due to lab-measurement errors. The missing twin pair was from the RATSS sample, concordant on no autism status, wherein one of the twins was diagnosed with ADHD. Since no discordance existed for autism diagnosis, and the pair were not unaffected from a psychiatric diagnosis, they were only used for the sensitivity analysis. The proteins in which both twins had too low fluorescence value to accurately calculate were CRP (2 pairs), SAA (3 pairs), SAP (5 pairs), and tPA (1 pair) – all pairs belong to the unaffected autism group. Hence for each APP-specific analysis, the child with missing values and their twin were both dropped from that specific analysis. ∗One family from the RATSS contributed triplets.

The two samples were combined into one for the main analysis in this study, with RATSS providing 135 twin pairs and SYC 86 twin pairs (Fig. 1). RATSS had full information on zygosity, whereas 18 outcome-concordant same-sex pairs identified within SYC lacked this information and were dropped from further analysis. To fulfill the primary aim of our study, we relied on twins without a neurodevelopmental condition and with known zygosity (Fig. 1). In total, 48 monozygotic and 44 dizygotic twin pairs were concordant in terms of having no neurodevelopmental condition (autism, intellectual disability [ID], or ADHD). For our secondary aim, we relied on twin pairs discordant for autism. Of the 60 autism-discordant twin families, one family had triplets, wherein one child was autistic – creating two “twin families” using the same autistic child together with each of the non-autistic triplets, resulting in 61 autism-discordant pairs (17 monozygotic pairs, 43 dizygotic pairs, and 1 pair with unknown zygosity).

### 2.2 Neurodevelopmental outcome assessment

Autism was ascertained in different ways depending on the source of data. For the RATSS data, clinical consensus diagnosis was made according to DSM-5 criteria corroborated the Swedish forms of Autism Diagnostic Interview – Revised (ADI-R) and the Autism Diagnostic Observation Schedule second edition (ADOS-2).

For SYC, all available pathways to care in Stockholm County, identified through population registers (Idring et al., 2012, 2015), were searched for autism diagnoses based on the F84 ICD-10 code (DSM-IV: 299). The searched registers included the National Patient Registers (both the inpatient and outpatient care, available since 1987 and 1997, respectively), the VAL Register (a Stockholm County register containing inpatient, outpatient, specialist, and primary health care data), and the Habilitation Register (information regarding use of habilitation services in Stockholm country – after 2008 this register was merged with the VAL register).

### 2.3 Collection of NDBS samples and analysis of acute phase proteins

All NDBS, originally collected as part of neonatal screening programs for metabolic disorders (known as the “PKU test”), were retrieved from the national biobank at Karolinska University Hospital in Solna, Sweden. Since 1981, all samples have been stored in a controlled environment at +4 °C and 30 % relative humidity. Following previously established protocols (Gardner et al., 2021), protein fractions were eluted from the NDBS samples and measured the concentration of 9 different APP in the eluates: procalcitonin (PCT), ferritin (FER), tissue plasminogen activator (tPA), fibrinogen (FIB), serum amyloid A (SAA), alpha-2 macroglobulin (A2M), C-reactive protein (CRP), haptoglobin (HAP) and serum amyloid P (SAP).

For the APP analyses, 3.2 mm diameter discs were punched from each NDBS and immersed in 200 μl of phosphate-buffered saline. The samples were agitated for 2 h at room temperature on a rotary shaker (600 rpm), put on ice and subsequently transferred to analytical plates. as previously described (Gardner et al., 2021). Single matched twin samples were processed on the same plate, side-by-side. Undiluted eluates were analyzed for procalcitonin, ferritin, tissue plasminogen activator, fibrinogen and serum amyloid A (PCT, FER, tPA, FIB and SAA, respectively), or were diluted 1:4 in sample diluent buffer provided by the manufacturer (Bio-Rad, Hercules, CA) for analyses of alpha-2 macroglobulin, C-reactive protein, haptoglobin and serum amyloid P (A2M, CRP, HAP and SAP, respectively) using premixed multiplex panels on the Bio-Plex 200 System (Bio-Rad). Two Negative controls (eluates from blank filter papers) and two positive controls were added to each analytical plate along with a standard curve according to the manufacturer's instructions (Bio-Rad). We used the emitted fluorescence, instead of the predicted concentration obtained by the included standard curves, for each biomarker to avoid imputations at the low end of detection. Doing this, we achieve a higher statistical power and lower coefficient of variation (Breen et al., 2015, 2016). In the event of both twins having fluorescence values below the limit of detection, as determined by the instrument and based on the standard curves, they were counted as missing due to uninformative comparisons. The proteins for which this occurred were CRP (2 pairs), SAA (3 pairs), SAP (5 pairs), and tPA (1 pair). One twin pair had a missing value for A2M, CRP, HAP, and SAP due to lab-measurement errors. For each specific analysis, the twin with missing values and their co-twin were dropped from that specific analysis (Fig. 1).

All APP fluorescence values were log_2_-transformed and standardized within their own plates, using the mean and standard deviation among unaffected twins.

### 2.4 Statistical analysis

The primary analytical goal of this study was to understand the relative contributions by genetic and environmental factors to variation in levels of APP in neonatal blood. To estimate the relative proportions of the different factors contributing to variation, additive genetic variation (A), common shared environment (C), and individual environment (E) for the twins’ APPs, we used models which compare monozygotic (MZ) and dizygotic (DZ) twins by modelling the shared genetic variation in twin pairs (100 % for MZ and 50 % for DZ twin pairs), as well as shared environmental variation (100 % in both MZ and DZ twin pairs) and non-shared environmental variation, in a structural equation model referred to as an ACE model. To test for potential dominant genetic effects (D), the ADE model was also estimated. The ACE and ADE model fits were compared against the AE model, to identify the best-fitting model.

To avoid sampling bias, we selected the MZ and DZ twin pairs without autism diagnosis (74 and 69 pairs, respectively) for ACE modeling (Fig. 1). We then excluded pairs that included a twin with ADHD (29 pairs) or ID diagnosis (5 pairs), leaving 48 dizygotic and 44 monozygotic pairs for analysis (Fig. 1). ACE/ADE models were thus estimated using twins with no missing data on the APPs, no known neurodevelopmental diagnosis and with known zygosity, controlling for the sex of each twin and the age at NDBS sample (days). We estimated the relative proportions for each of the APPs using the ACE/ADE models with structural equation modelling from the mets R package (Holst et al., 2016; Scheike et al., 2014). To find the most statistically optimal model fit, we then tested the lowest number of estimated parameters against the others (e.g., ACE versus AE or ADE versus AE).

A subgoal of our study was to test whether APPs at the time of birth were associated with children's autism. We selected the twin pairs in which only one twin had an autism diagnosis (61 pairs from 60 families, Fig. 1). We first estimated the models with a standard logistic regression model, adjusting for birth year, zygosity status, sex, and age at sample (days). The standard logistic regression model is, in this scenario, considered a *between-pairs estimate*. Second, we accounted for the shared genetic and environmental backgrounds of the twin pair analyses by using fixed-effects logistic regression models (*within-pairs estimate*) (Sjölander et al., 2022), conditioning on the maternal identity and adjusting for sex and age at sample.

We stratified the twin types into MZ and DZ groups for the autism analyses and compared the differences between the zygosity groups.

All regression models were estimated using cluster robust standard errors conditioned on the family (Sjölander et al., 2022). All confidence intervals are calculated with a 95 % threshold. Bonferroni corrections for multiple tests were calculated, as the limited sample sizes and numerous tests increases the risk of false-positive findings. The Bonferroni threshold was set to the significant level of 0.005 (0.05/9, rounded down), based on the nine APPs being analyzed.

The data management and regression analyses were performed in StataMP 17, and the ACE/ADE models in R version 4.2.3.

## 3 Results

### 3.1 Study population

The unaffected twin pairs had similar zygosity distributions (44.0 % MZ, and 44.7 % DZ), but the autism discordant pairs had notably fewer MZ pairs (27.9 %), compared to DZ pairs (70.5 %) (Table 1). ID and ADHD were more common in twin pairs where at least one twin was also diagnosed with autism. The age at NDBS sampling was 4.1 days, and did not differ between the MZ and DZ pairs (Table 2) (p-value: 0.079), between autism discordant twins (p-value: 0.27) (Table A1), or depending on neurodevelopmental condition (Table A2).

**Table 1:** *Descriptive information of the distribution and availability of twin pair data. Stratified on autism status within the pairs (Unaffected: No autistic twin, Discordant: One autistic twin, Concordant: Both twins autistic) against the categorical factors. The given p-value is the chi-square test. ID=Intellectual Disability.* IQR= Interquartile range.

		Autism status			p-value
		Unaffected	Discordant	Concordant	
N (twin pairs)		141	61	20	
**Number of females**	*No females*	52 (36.9 %)	29 (47.5 %)	14 (70.0 %)	0.009
	*1 female*	23 (16.3 %)	15 (24.6 %)	1 (5.0 %)	
	*Both female*	66 (46.8 %)	17 (27.9 %)	5 (25.0 %)	
**ADHD distribution**	*Unaffected*	112 (79.4 %)	37 (60.7 %)	8 (40.0 %)	<0.001
	*Discordant*	18 (12.8 %)	17 (27.9 %)	6 (30.0 %)	
	*Concordant*	11 (7.8 %)	7 (11.5 %)	6 (30.0 %)	
**ID distribution**	*Unaffected*	136 (96.5 %)	43 (70.5 %)	12 (60.0 %)	<0.001
	*Discordant*	5 (3.5 %)	17 (27.9 %)	4 (20.0 %)	
	*Concordant*	0 (0.0 %)	1 (1.6 %)	4 (20.0 %)	
**Zygosity type**	*Monozygotic twins*	62 (44.0 %)	17 (27.9 %)	12 (60.0 %)	0.002
	*Dizygotic twins*	63 (44.7 %)	43 (70.5 %)	6 (30.0 %)	
	*Unknown zygosity*	16 (11.3 %)	1 (1.6 %)	2 (10.0 %)	
**Birthyear**	*Median (IQR)*	1998 (1996, 2000)	1998 (1996, 2000)	1998 (1996, 2000)

**Table 2:** Descriptive information about the neonatal acute phase proteins for the included twin pairs in the ACE/ADE models, stratified on zygosity. Selected with the criteria of being without any autism, ADHD or intellectual disability (ID) diagnosis. Shown p-values are from unpaired t-tests, examining the difference in distribution averages of APPs between the dizygotic and monozygotic twins. Variation in the number of pairs is due to both twins being below the limit of detection and therefore became excluded from the analysis.

Sample for the ACE/ADE models	Number of pairs	Dizygotic twins	Number of pairs	Monozygotic twins	p-value
**A2M, mean (SD)**	48	−0.204 (0.974)	44	−0.052 (1.208)	0.34
**CRP, mean (SD)**	46	−0.186 (0.883)	44	0.150 (1.072)	0.023
**FER, mean (SD)**	48	−0.041 (0.939)	44	−0.169 (1.144)	0.41
**FIB, mean (SD)**	48	−0.174 (1.075)	44	−0.041 (1.110)	0.41
**HAP, mean (SD)**	48	−0.121 (1.096)	44	0.267 (1.081)	0.017
**PCT, mean (SD)**	48	−0.095 (1.061)	44	−0.018 (0.897)	0.60
**SAA, mean (SD)**	48	−0.201 (0.814)	42	0.358 (1.607)	0.003
**SAP, mean (SD)**	45	−0.140 (0.819)	43	0.035 (1.194)	0.26
**tPA, mean (SD)**	48	−0.191 (1.073)	43	−0.126 (1.073)	0.69
**Age at sample (days), mean (SD)**	48	4.12 (1.29)	43	3.89 (0.99)	0.079

### 3.2 APP correlations and heritability estimates

**Fig. 2:**
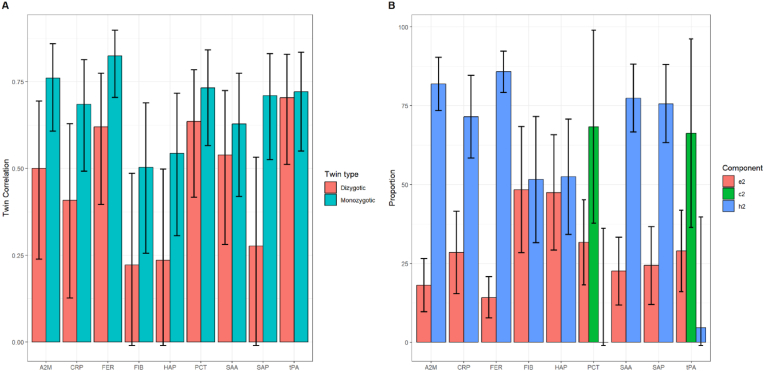
Within-twin pair Pearson's correlation stratified on twin status for unaffected dizygotic (48 pairs) and monozygotic (44 pairs) twins, and calculated proportions from the most statistically optimal ACE/AE model for the acute phase proteins. “e2” is the individual environment, “c2” the common shared environment, and “h2” is the proportion explained by additive genetic heritability. The ACE/AE models were adjusted for sex and age-at-NDBS-sample (in days). Each correlation was calculated alongside a 95 % confidence interval. A2M, a-2-macroglobulin; CRP, C-reactive protein; FER, ferritin; FIB, fibrinogen; HAP, haptoglobin; PCT, procalcitonin; SAA, serum amyloid A; SAP, serum amyloid P; tPA, tissue plasminogen activator.

Comparing MZ twin pairs to DZ twin pairs, CRP, HAP and SAA differed in their distributions (p-values: 0.023, 0.017, and 0.003, respectively), with the MZ twins having higher levels compared to DZ (Table 2). Within MZ pairs, all APP were significantly positively correlated, ranging between 0.503 (0.256, 0.689) for FIB, and 0.824 (0.705, 0.898) for FER (Fig. 2, Table A3). All APPs, except FIB, HAP and SAP, were also significantly positively correlated within DZ twin pairs – all DZ within-pair correlations were either equal to or smaller than the corresponding MZ correlations (Fig. 2, Table A3).

A2M, FER, SAA and SAP had heritability estimates of over 75 % in the ACE models. PCT and tPA had significant contributions from the shared environment component to their variations, 68.3 % (33.7; 98.9) and 66.3 % (36.4; 96.2), respectively, but no statistically significant contribution from heritability (0 % [−36.1; 36.2] and 4.6 % [−30.5; 39.7], respectively). There was no strong evidence of any contributions from the shared environment to the variations of A2M, CRP, FER, FIB, SAA, SAP, and HAP (Fig. 2, Table A4). Traces of a dominant genetic effect were observed for SAA and FIB, though the ADE models did not statistically improve the model fits compared to the AE model (Table A4).

The ACE model tests of equal variances and averages within and between twin pairs and zygosity, for the APPs, yielded different results. Not all assumptions for ACE models were met for all APP tested- A2M, CRP, HAP, SAA, and SAP were statistically significant for the between-zygosity-types *t*-test for mean differences (Table A5). SAP did not fulfill the equal variance between zygosity type assumption (p-value: 0.010). PCT and SAA broke the assumption of equal variance test across twin order within both the monozygotic (p-value: 0.019 and 0.013, respectively) and dizygotic groups (p-value: 0.041 and < 0.001, respectively), indicating that their robustness in estimation should be treated with some caution. The proteins which fulfilled all ACE/ADE assumptions were FER, FIB and tPA.

### 3.3 Associations between APP and neurodevelopmental outcomes

The average age for autism diagnosis was 6 years (SD = 2.8) in the SYC sample, and 9 years (SD = 4.1) in RATSS sample. For the autism-discordant sample, no association was observed between mean levels of any of the 9 APPs and autism, using a paired *t*-test (Table A1).

There were no clear associations between APP levels and autism, regardless of zygosity or regression model conditioning (Table A4). In general, the point estimates were heightened for most APP levels, but there existed no clear pattern of attenuation for either the zygosity steps or when conditioning on the twins.

For potential sample source differences and their relationship with odds of autism, both FER and PCT showed some differences, with the RATSS samples trending towards a potential protective effect for FER and risk for PCT, with the SYC sample showing the opposite patterns (Table A7).

## 4 Discussion

This is the first study, to our knowledge, to examine the relative contributions of heritable and environmental factors to the variation in neonatal APP levels. We observed a notable contribution of heritable components, explaining more than 50 % of the variation in the levels of A2M, CRP, FER, SAA and SAP. More subtle contributions to the variation by heritable factors were observed for FIB and HAP, while substantial contributions to the variation by shared environmental factors were observed for PCT and tPA. We additionally attempted to examine the association between neonatal levels of APP and autism in this unique, but limited, sample of autism-discordant twin pairs, but our results were largely inconclusive, likely due to a lack of statistical power.

### 4.1 Heritability of APP

A previous study estimated the heritability for CRP and SAA in adults, with estimates of 52 % for CRP and 59 % for SAA (MacGregor et al., 2004). Those estimates are substantially lower than our current estimates obtained within a few days of birth of 72 % and 77 % for CRP and SAA, respectively. Similarly, a heritability of FER levels among adult female twins of 44 % was previously reported (Fairweather-Tait et al., 2013), compared to 86 % observed here. Another study looking at FIB and tPA reported heritability estimates of 39 % and 67 % among adults (de Lange et al., 2006), indicating an estimate for FIB similar to our estimate (52 %) but different for tPA, as we did not find evidence of any contribution from heritability in our samples taken at the time of birth.

We could not find any twin-based studies using ACE models in the literature for A2M, HAP, PCT and SAP. Although polymorphisms associated with their levels have previously been identified (Suhre et al., 2017; Sun et al., 2018), the relative influences of heritable and environmental factors on their levels (in neonatal blood) reported here are novel. Zhang et al. (2025) recently identified three genomic loci contributing to the variation in PCT levels in the first GWAS of circulating levels of PCT in adult individuals.

Variations in CRP, SAA and FER appear to be more heritable at birth compared to later in life. Interestingly, in a large longitudinal twin-study, Sas and co-workers reported stable contributions from genetic contributions in CRP over a 10-year period (Sas et al., 2017) – showing potential evidence of the heritability only differing in the early ages, and then become constant. FIB appears stable from birth to adulthood, whereas the variation in tPA appears to be less explained by heritable factors at birth compared to later in life. It should be noted that all studies which have examined the variation in these APPs using ACE models used sera taken years after birth, not uncommonly from heterogeneous groups with a wide age-range at sampling (MacGregor et al., 2004; Fairweather-Tait et al., 2013; de Lange et al., 2006).

A higher heritability of APP at the time of birth compared to later years, as observed here for CRP, SAA and FER, might well be anticipated since adults have a considerably longer history of environmental exposures influencing their levels compared to neonates. Increased diversity with aging has also been noted for other aspects of immune functioning measured in monozygotic twins (Brodin et al., 2015; Yan et al., 2021), indicating that the influences of non-heritable factors often increase with age. With restricted windows of environmental exposures at the time of birth, and the ACE model being an estimate of proportions, it is not surprising that environmental exposures would contribute a larger proportion of its variation later in life. The approximate zero heritability of tPA and PCT among newborns was therefore surprising, but it may support their use as indicators of environmental influences in the perinatal period (van Rossum et al., 2004; Dandona et al., 1994).

The importance of understanding the different sources of variation in APP levels throughout life is illustrated by recent studies supporting a causal role of CRP levels in schizophrenia, cancer, and cardiovascular disease (Said et al., 2022) along with evidence associating immune system variation during early life with autoimmune and inflammatory conditions later in life (Lee et al., 2022). To understand how levels of CRP cause these diverse conditions, future large-scale studies ought to consider the contributions of both genetic and environmental factors to its variation – either by large-scale twin or with genetic studies complemented by deep phenotypic characterization.

### 4.2 APP and neurodevelopmental outcomes

Yin, Wang (Yin et al., 2020) combined several smaller clinical studies examining CRP levels, measured around age four, and autism and concluded that autistic children have higher levels of CRP. Those studies were based on cross-sectional measurements and could not rule out the potential for a reverse causation. Furthermore, control of genetic factors that might influence CRP levels was incomplete and none of the studies accounted for familial factors. A Danish study analyzed neonatal CRP levels in a case-control design and found no association with autism (Skogstrand et al., 2019). We previously reported that CRP displayed a u-shaped association with odds of autism in a case-control design, but the association was not significant when comparing siblings (Gardner et al., 2021). In this study, we found no evidence of an association between CRP and autism. This is in line with Mendelian Randomization studies observing no causal role of CRP in autism (Reay et al., 2022; Prins et al., 2016).

### 4.3 Strengths and limitations

The strengths of this study are several. It is the biggest twin study to date that estimates the genetic and environmental influence of the APPs – some of them which, like PCT, had no previous estimates available. The study is also unique in that the markers were measured just a few days after birth for all children. Even if this is one of the larger molecular twin studies, the limited sample size still needs to be taken into consideration. No stratification was made based on sex, even though hypothesized sex differences in the early-life immune system exist (Kim et al., 2023; Schlinzig et al., 2017). We adjusted for the children's sex in the ACE/ADE models, but acknowledge that potential interaction effects could be an issue. Another limitation is that A2M, CRP, HAP, PCT, SAA and SAP violated the ACE/ADE model assumptions, rendering their estimates potentially biased.

Autism has a higher prevalence in males than females. Males' early-life immune system is also different from females’ (Kim et al., 2023; Schlinzig et al., 2017), increasing the risk of heterogeneity in the associations of neonatal APPs and later odds of autism based on sex. Even though we aimed to capture as much shared intrauterine environments as possible by comparing twins, there may still be differences, e.g., twins can compete for resources while in utero, which may contribute to increased individual differences which we were unable to control or examine in this study.

SYC used register-based sampling and RATSS used an open recruitment-based design. This could lead to potential differences in each study's ability to ascertain cases. RATSS may potentially capture milder phenotypes, while SYC captured only diagnosed cases, potentially reflecting the more severe cases. Since such differences could bias the outcome ascertainment in opposite directions, combining the two samples could then help to balance out any biases introduced by these sampling strategies. Another risk related to biased sampling could be due to the exclusion of autism-affected individuals in the AE/ACE models, leaving us with a potentially “healthier” sample. However, due to both the low prevalence of autism in the general population and apparent non-association with APPs, the selection-bias introduced by focusing on non-autistic twin pairs is likely negligible.

## 5 Conclusions

The variation in most of the APPs, measured shortly after birth in this study, showed a notable and strong contribution from heritable factors. Given that A2M, CRP, FER, FIB, HAP, SAA, and SAP had optimal fits with AE models, there is little evidence that shared maternal environmental factors during pregnancy have major influences on these early life APP levels. The hereditary proportions we identified were larger among neonates for most APP, compared to those calculated when APP were measured later in life. There were predominant environmental influences on the variations of tPA and PCT in the neonatal period. Larger twin studies are required to confirm these conclusions, with special emphasis on homogeneity in terms of the age of the twins at the time of measurement in the sampling design. We found no evidence of APP levels at birth might be associated with autism, yet the small sample size here prohibits any firm conclusions.

## CRediT authorship contribution statement

**Hugo Sjöqvist:** Conceptualization, Formal analysis, Software, Writing – original draft, Data curation, Methodology, Visualization, Writing – review & editing. **Martin Brynge:** Data curation, Writing – review & editing, Investigation. **Kristiina Tammimies:** Writing – review & editing, Investigation. **Ralf Kuja-Halkola:** Writing – review & editing, Methodology. **Sven Bölte:** Investigation, Writing – review & editing, Funding acquisition, Resources. **Christina Dalman:** Funding acquisition, Project administration, Writing – original draft, Conceptualization, Investigation, Supervision, Writing – review & editing. **Renee M. Gardner:** Conceptualization, Funding acquisition, Methodology, Supervision, Writing – review & editing, Data curation, Investigation, Project administration, Writing – original draft. **Håkan Karlsson:** Funding acquisition, Methodology, Supervision, Writing – review & editing, Conceptualization, Investigation, Resources, Writing – original draft.

## Funding

This work was supported by grants from the 10.13039/501100004359Swedish Research Council (Grant Nos. 523-2009-7054, 521-2010-4409, 259-2012-24, 521-2013-2531 and 2017-00641 [to SB]; 2017–02900 and 2022-00592 [to RMG]; 523–2010-1052 and 2021-01306 [to 10.13039/100011639CD]; and 2018–02907 [to HK]), Hjärnfonden grant (Grant No. FO2012-0245, FO2013-0163, FOU2014-0228 and FO2015-0095 [to SB]; FO2020-0019 [to HK]; FO2023-0266 [to RMG]), 10.13039/100007123Stanley Medical Research Institute (Grant No. NV-002 [to HK]) and 10.13039/501100001034ALF Medicine (Grant No. 20100096, 20110602, 20140134 and 20170016 [to SB]). Additionally, the following [to SB] was used to fund this study: Foundation Sunnerdahls Handikappfond (7/11), Stockholm Brain Institute (Berzelli Award, 2012), Swedish Order of Freemasons (2014-06-14 and 2017-05-23), Sällskapet Barnavård, Queen Silvia Jubilee Fund, 10.13039/100010823Tore Nilsson Foundation (2012-12-10), 10.13039/501100006715Solstickan Foundation, PRIMA Child and Adult Psychiatry, The Pediatric Research Foundation at Astrid Lindgren Children's Hospital, Jerring Foundation, 10.13039/501100009447The Kempe-Carlgrenska Foundation. SB also received support from the EU/10.13039/100013322EFPIA/10.13039/100014370SFARI/Autistica/AUTISM SPEAKS 10.13039/501100010767Innovative Medicines Initiative 2 Joint Undertaking (AIMS-2-TRIALS Grant no 777394). Any views expressed are those of the author(s) and not necessarily those of the funders.

The Swedish Twin Registry is managed by 10.13039/501100004047Karolinska Institutet and receives funding through the 10.13039/501100004359Swedish Research Council under the grant no 2017-00641.

None of these sponsors had any role in the writing of this article.

## Declaration of competing interest

The authors declare that they have no known competing financial interests or personal relationships that could have appeared to influence the work reported in this paper.
