# Supplementary material for: Heritability of neonatal acute phase protein levels
Source: Brain Behav Immun Health. 2025 Sep 12;49:101097. doi: 10.1016/j.bbih.2025.101097 (PMC12466238; doi:10.1016/j.bbih.2025.101097)
Supplement: Multimedia component 1 [file mmc1.docx]

| Sample for the autism analysis | **Number of pairs** | **No Autism** | **Autism** | **p-value** |
| --- | --- | --- | --- | --- |
| **A2M, mean (SD)** | 61 | 0.032 (0.91) | 0.030 (1.15) | 0.99 |
| **CRP, mean (SD)** | 61 | -0.057 (0.97) | 0.015 (1.01) | 0.65 |
| **FER, mean (SD)** | 61 | 0.017 (1.00) | 0.028 (1.08) | 0.91 |
| **FIB, mean (SD)** | 61 | -0.021 (0.99) | 0.054 (1.05) | 0.60 |
| **HAP, mean (SD)** | 61 | 0.043 (0.93) | 0.116 (1.13) | 0.60 |
| **PCT, mean (SD)** | 61 | 0.091 (1.07) | 0.486 (2.16) | 0.16 |
| **SAA, mean (SD)** | 61 | 0.028 (0.95) | 0.106 (1.48) | 0.70 |
| **SAP, mean (SD)** | 61 | -0.031 (1.09) | 0.085 (0.91) | 0.40 |
| **tPA, mean (SD)** | 61 | 0.037 (0.95) | 0.249 (1.33) | 0.21 |
| **Age at sample (days),  mean (SD)** | 61 | 4.28 (2.40) | 4.53 (2.77) | 0.27 |

**Table A1.** Autism-discordant twin pairs alongside their associations with the neonatal acute phase proteins. Shown p-values are from the paired t-test within the twin pairs.
A2M, a-2-macroglobulin; CRP, C-reactive protein; FER, ferritin; FIB, fibrinogen; HAP, haptoglobin; PCT, procalcitonin; SAA, serum amyloid A; SAP, serum amyloid P; tPA, tissue plasminogen activator.

|  | **Unaffected individuals** | **Any  autism** | **p-value** | **Any  ADHD** | **p-value** | **Any  ID** | **p-value** |
| --- | --- | --- | --- | --- | --- | --- | --- |
| **N** | 291 | 101 |  | 88 |  | 36 |  |
| **Autism** | 0 (-) |  |  | 41 (46.6%) |  | 29 (80.6%) |  |
| **ADHD** | 0 (-) | 41 (40.6%) |  |  |  | 13 (36.1%) |  |
| **ID** | 0 (-) | 29 (28.7%) |  | 13 (14.8%) |  |  |  |
| **Female** | 162 (55.7%) | 32 (31.7%) | <0.001 | 28 (31.8%) | <0.001 | 13 (36.1%) | 0.026 |
| **A2M,  mean (SD)** | -0.062 (0.998) | 0.079 (1.068) | 0.23 | 0.202 (1.076) | 0.034 | 0.188 (0.732) | 0.15 |
| **CRP,  mean (SD)** | -0.014 (0.981) | 0.093 (0.975) | 0.35 | 0.262 (0.889) | 0.019 | 0.293 (0.870) | 0.073 |
| **FER,  mean (SD)** | -0.044 (1.016) | 0.092 (0.988) | 0.24 | 0.183 (0.858) | 0.058 | 0.037 (1.058) | 0.65 |
| **FIB,  mean (SD)** | -0.080 (1.080) | 0.019 (1.016) | 0.42 | 0.082 (0.978) | 0.21 | 0.165 (0.879) | 0.19 |
| **HAP,  mean (SD)** | 0.026 (0.988) | 0.015 (1.064) | 0.92 | 0.165 (1.048) | 0.26 | -0.117 (1.160) | 0.42 |
| **PCT,  mean (SD)** | -0.009 (1.011) | 0.314 (1.836) | 0.029 | 0.396 (1.705) | 0.006 | 0.063 (1.076) | 0.69 |
| **SAA,  mean (SD)** | 0.021 (1.156) | 0.250 (1.448) | 0.11 | 0.223 (1.304) | 0.17 | 0.336 (1.849) | 0.16 |
| **SAP,  mean (SD)** | -0.026 (0.913) | 0.090 (0.992) | 0.28 | 0.237 (0.908) | 0.020 | 0.246 (0.853) | 0.091 |
| **tPA,  mean (SD)** | -0.098 (1.031) | 0.274 (1.313) | 0.004 | 0.319 (1.366) | 0.002 | 0.174 (1.279) | 0.15 |
| **Age at sample (days),  mean (SD)** | 4.131 (1.588) | 4.356 (2.274) | 0.28 | 3.852 (1.160) | 0.13 | 4.139 (1.175) | 0.98 |

**Table A2.** Descriptive sample distribution on the individual level, stratified on neurodevelopmental condition status. Shown p-values are from chi2 and t-tests between unaffected group and each neurodevelopmental outcome group. The affected groups are not mutually exclusive, but one individual can belong to all three, given the individual has all three diagnoses.
A2M, a-2-macroglobulin; CRP, C-reactive protein; FER, ferritin; FIB, fibrinogen; HAP, haptoglobin; PCT, procalcitonin; SAA, serum amyloid A; SAP, serum amyloid P; tPA, tissue plasminogen activator.

|  | **Dizygotic twins** | **Monozygotic twins** |
| --- | --- | --- |
| **A2M, correlation (95% CI)** | 0.500 (0.239, 0.694) | 0.760 (0.608, 0.859) |
| **CRP, correlation (95% CI)** | 0.408 (0.127, 0.629) | 0.685 (0.492, 0.813) |
| **FER, correlation (95% CI)** | 0.620 (0.396, 0.775) | 0.824 (0.705, 0.898) |
| **FIB, correlation (95% CI)** | 0.221 (-0.081, 0.486) | 0.503 (0.256, 0.689) |
| **HAP, correlation (95% CI)** | 0.236 (-0.007, 0.498) | 0.543 (0.307, 0.717) |
| **PCT, correlation (95% CI)** | 0.635 (0.417, 0.784) | 0.732 (0.565, 0.841) |
| **SAA, correlation (95% CI)** | 0.539 (0.281, 0.724) | 0.628 (0.419, 0.774) |
| **SAP, correlation (95% CI)** | 0.276 (-0.026, 0.532) | 0.710 (0.526, 0.830) |
| **tPA, correlation (95% CI)** | 0.704 (0.511, 0.829) | 0.721 (0.550, 0.834) |

**Table A3.** Within-twin correlations (with 95% CI=confidence interval) for the neonatal acute phase proteins, stratified on zygosity. Restricted sample to the ACE/ADE models – with selection criteria of being without any autism, ADHD or intellectual disability (ID) diagnosis.

| **APP** | **Type** | **AE** | **ACE** | **ADE** |
| --- | --- | --- | --- | --- |
| **A2M** | A | ***81.9 (73.5; 90.3)** | 71.3 (30.7; 111.8) | 81.9 (73.5; 90.3) |
|  | C | *NA* | 10.5 (-28.5; 49.4) | *NA* |
|  | D | *NA* | *NA* | 0 (0; 0) |
|  | E | ***18.1 (9.7; 26.5)** | 18.2 (9.5; 26.9) | 18.1 (9.7; 26.5) |
| **CRP** | A | ***71.5 (58.4; 84.6)** | 70 (19.5; 120.4) | 71.5 (58.4; 84.6) |
|  | C | *NA* | 1.5 (-44.8; 47.7) | *NA* |
|  | D | *NA* | *NA* | 0 (0; 0) |
|  | E | ***28.5 (15.4; 41.6)** | 28.6 (15.2; 41.9) | 28.5 (15.4; 41.6) |
| **FER** | A | ***85.8 (79.2; 92.3)** | 60.7 (24.1; 97.3) | 85.8 (79.2; 92.3) |
|  | C | *NA* | 25 (-10.9; 60.9) | *NA* |
|  | D | *NA* | *NA* | 0 (0; 0) |
|  | E | ***14.2 (7.7; 20.8)** | 14.3 (7.4; 21.2) | 14.2 (7.7; 20.8) |
| **FIB** | A | ***51.6 (31.6; 71.6)** | 51.6 (31.6; 71.6) | 28.2 (-77.5; 134) |
|  | C | *NA* | 0 (0; 0) | *NA* |
|  | D | *NA* | *NA* | 24.9 (-85.4; 135.2) |
|  | E | ***48.4 (28.4; 68.4)** | 48.4 (28.4; 68.4) | 46.9 (26.8; 66.9) |
| **HAP** | A | ***52.5 (34.2; 70.8)** | 52.5 (34.2; 70.8) | 51.3 (-60; 162.5) |
|  | C | *NA* | 0 (0; 0) | *NA* |
|  | D | *NA* | *NA* | 1.3 (-113.4; 116) |
|  | E | ***47.5 (29.2; 65.8)** | 47.5 (29.2; 65.8) | 47.4 (28.5; 66.4) |
| **PCT** | A | 69.7 (58.2; 81.3) | ***0 (-36.1; 36.2)** | 69.7 (58.2; 81.3) |
|  | C | *NA* | ***68.3 (37.7; 98.9)** | *NA* |
|  | D | *NA* | *NA* | 0 (0; 0) |
|  | E | 30.3 (18.7; 41.8) | ***31.7 (18.2; 45.2)** | 30.3 (18.7; 41.8) |
| **SAA** | A | ***77.4 (66.7; 88.2)** | 68.2 (25.5; 110.9) | 77.4 (66.7; 88.2) |
|  | C | *NA* | 8.8 (-30.6; 48.3) | *NA* |
|  | D | *NA* | *NA* | 0 (0; 0) |
|  | E | ***22.6 (11.8; 33.3)** | 22.9 (11.6; 34.3) | 22.6 (11.8; 33.3) |
| **SAP** | A | ***75.6 (63.3; 88)** | 75.6 (63.3; 88) | 25.1 (-72.9; 123.1) |
|  | C | *NA* | 0 (0; 0) | *NA* |
|  | D | *NA* | *NA* | 51.5 (-47.5; 150.4) |
|  | E | ***24.4 (12; 36.7)** | 24.4 (12; 36.7) | 23.4 (12.2; 34.7) |
| **tPA** | A | 72.5 (61.7; 83.3) | ***4.6 (-30.5; 39.7)** | 72.5 (61.7; 83.3) |
|  | C | *NA* | ***66.3 (36.4; 96.2)** | *NA* |
|  | D | *NA* | *NA* | 0 (0; 0) |
|  | E | 27.5 (16.7; 38.3) | ***29 (16.1; 41.9)** | 27.5 (16.7; 38.3) |

**Table A4.** Predicted proportions of the ACE/ADE models, along with their 95% confidence interval for each acute phase protein (APP).
NA=Not Available. All models were adjusted for sex and age-at-PKU sample (measured in number of days).
* This model was the most statistically optimal (significance level 10%) out of all three.
A2M, a-2-macroglobulin; CRP, C-reactive protein; FER, ferritin; FIB, fibrinogen; HAP, haptoglobin; PCT, procalcitonin; SAA, serum amyloid A; SAP, serum amyloid P; tPA, tissue plasminogen activator.

| **APP** | **t-test_Zygosity_** | **Var_Zygosity_** | **t-test_Mono_** | **Var_Mono_** | **t-test_Dizygotic_** | **Var_Dizygotic_** |
| --- | --- | --- | --- | --- | --- | --- |
| **A2M** | **<0.001** | 0.122 | 0.765 | 0.475 | 0.498 | 0.237 |
| **CRP** | **<0.001** | 0.879 | 0.756 | 0.716 | 0.280 | 0.837 |
| **FER** | 0.021 | 0.176 | 0.940 | 0.314 | 0.802 | 0.262 |
| **FIB** | 0.095 | 0.162 | 0.544 | 0.511 | 0.875 | 0.896 |
| **HAP** | **0.007** | 0.347 | 0.613 | 0.812 | 0.741 | 0.116 |
| **PCT** | 0.431 | 0.798 | 0.193 | **0.019** | 0.577 | **0.041** |
| **SAA** | **0.015** | 0.061 | 0.359 | **0.013** | 0.168 | **<0.001** |
| **SAP** | **<0.001** | **0.010** | 0.737 | 0.635 | 0.488 | 0.644 |
| **tPA** | 0.150 | 0.132 | 0.616 | 0.141 | 0.315 | 0.207 |

**Table A5.** P-values of the tests for the ACE/ADE models for each acute phase protein (APP). “t-test” and “Var” corresponds to a test of equal means and equal variances. “Zygosity” is a test between the monozygotic and dizygotic twins. “Mono” and “Dizygotic” compare the within-twin-pair differences for each zygosity group.
A2M, a-2-macroglobulin; CRP, C-reactive protein; FER, ferritin; FIB, fibrinogen; HAP, haptoglobin; PCT, procalcitonin; SAA, serum amyloid A; SAP, serum amyloid P; tPA, tissue plasminogen activator.

| **APP** | **Zygosity** | **Adjusted non-fixed effects model** | **Adjusted fixed effects model** |
| --- | --- | --- | --- |
| **A2M** | Dizygotic | 1.23 (0.82; 1.86) | 1.52 (0.69; 3.30) |
|  | Monozygotic | 0.87 (0.61; 1.23) | 0.78 (0.39; 1.55) |
| **CRP** | Dizygotic | 1.06 (0.74; 1.51) | 1.04 (0.60; 1.79) |
|  | Monozygotic | 1.01 (0.64; 1.59) | 1.09 (0.55; 2.17) |
| **FER** | Dizygotic | 1.17 (0.85; 1.63) | 0.92 (0.37; 2.27) |
|  | Monozygotic | 1.05 (0.67; 1.65) | 5.35 (0.13; 216.10) |
| **FIB** | Dizygotic | 1.20 (0.88; 1.63) | 1.53 (0.81; 2.89) |
|  | Monozygotic | 0.97 (0.71; 1.34) | 0.91 (0.45; 1.85) |
| **HAP** | Dizygotic | 1.01 (0.71; 1.44) | 0.96 (0.49; 1.88) |
|  | Monozygotic | 0.98 (0.64; 1.51) | 1.20 (0.51; 2.80) |
| **PCT** | Dizygotic | 1.24 (0.99; 1.55) | 1.15 (0.79; 1.67) |
|  | Monozygotic | 1.13 (0.85; 1.52) | 2.25 (0.63; 8.10) |
| **SAA** | Dizygotic | 1.13 (0.87; 1.48) | 0.97 (0.62; 1.51) |
|  | Monozygotic | 1.45 (0.99; 2.12) | 0.76 (0.30; 1.91) |
| **SAP** | Dizygotic | 1.14 (0.79; 1.63) | 1.42 (0.73; 2.77) |
|  | Monozygotic | 0.86 (0.56; 1.33) | 1.00 (0.52; 1.95) |
| **tPA** | Dizygotic | 1.39 (1.01; 1.92) | 1.40 (0.79; 2.48) |
|  | Monozygotic | 1.21 (0.91; 1.63) | 1.78 (0.79; 3.98) |
| **Table A6.** Estimates with 95% confidence intervals for the odds ratios from the adjusted non-fixed effects logistic regression model, and the adjusted logistic regression model, examining the associations between the acute phase proteins (APPs) and autism. Stratifying on twin zygosity.  A2M, a-2-macroglobulin; CRP, C-reactive protein; FER, ferritin; FIB, fibrinogen; HAP, haptoglobin; PCT, procalcitonin; SAA, serum amyloid A; SAP, serum amyloid P; tPA, tissue plasminogen activator. | | | |

| **APP** | **Sample** | **Adjusted non-fixed effects model** | **Adjusted fixed effects model** |
| --- | --- | --- | --- |
| **A2M** | Combined | 0.99 (0.77; 1.26) | 1.00 (0.61; 1.65) |
|  | RATSS | 0.80 (0.60; 1.06) | 0.91 (0.52; 1.60) |
|  | SYC | 1.41 (0.75; 2.64) | 1.54 (0.50; 4.73) |
|  |  |  |  |
| **CRP** | Combined | 1.06 (0.80; 1.39) | 1.06 (0.69; 1.62) |
|  | RATSS | 0.94 (0.67; 1.32) | 1.04 (0.63; 1.71) |
|  | SYC | 1.17 (0.72; 1.91) | 1.10 (0.43; 2.84) |
|  |  |  |  |
| **FER** | Combined | 1.12 (0.87; 1.46) | 1.19 (0.56; 2.52) |
|  | RATSS | 0.78 (0.53; 1.14) | 0.73 (0.30; 1.77) |
|  | SYC | 1.66 (1.09; 2.51) | 5.79 (0.95; 35.14) |
|  |  |  |  |
| **FIB** | Combined | 1.03 (0.83; 1.29) | 1.13 (0.70; 1.84) |
|  | RATSS | 0.97 (0.75; 1.25) | 1.06 (0.62; 1.84) |
|  | SYC | 1.21 (0.79; 1.85) | 1.43 (0.49; 4.17) |
|  |  |  |  |
| **HAP** | Combined | 0.91 (0.70; 1.18) | 1.06 (0.64; 1.78) |
|  | RATSS | 1.00 (0.71; 1.42) | 1.29 (0.73; 2.30) |
|  | SYC | 0.74 (0.49; 1.12) | 0.30 (0.04; 2.22) |
|  |  |  |  |
| **PCT** | Combined | 1.16 (0.96; 1.40) | 1.24 (0.86; 1.79) |
|  | RATSS | 1.08 (0.87; 1.35) | 2.52 (1.21; 5.26) |
|  | SYC | 1.31 (0.75; 2.29) | 0.63 (0.25; 1.56) |
|  |  |  |  |
| **SAA** | Combined | 1.17 (0.96; 1.42) | 0.91 (0.59; 1.38) |
|  | RATSS | 1.03 (0.76; 1.41) | 0.93 (0.58; 1.51) |
|  | SYC | 1.25 (0.96; 1.65) | 0.70 (0.25; 1.96) |
|  |  |  |  |
| **SAP** | Combined | 0.98 (0.72; 1.33) | 1.19 (0.73; 1.95) |
|  | RATSS | 0.82 (0.59; 1.14) | 1.16 (0.69; 1.96) |
|  | SYC | 1.33 (0.71; 2.47) | 1.76 (0.29; 10.75) |
|  |  |  |  |
| **tPA** | Combined | 1.31 (1.04; 1.65) | 1.46 (0.92; 2.33) |
|  | RATSS | 1.08 (0.83; 1.40) | 2.03 (1.12; 3.67) |
|  | SYC | 2.01 (1.11; 3.67) | 0.83 (0.31; 2.20) |
| **Table A7.** Estimates with 95% confidence intervals for the odds ratios from the adjusted non-fixed effects logistic regression model, and the adjusted logistic regression model, examining the associations between the acute phase proteins (APPs) and autism. Combining and stratifying on sample source.  A2M, a-2-macroglobulin; CRP, C-reactive protein; FER, ferritin; FIB, fibrinogen; HAP, haptoglobin; PCT, procalcitonin; SAA, serum amyloid A; SAP, serum amyloid P; tPA, tissue plasminogen activator. | | | |
